# FAST Pre-Filtering-Based Real Time Road Sign Detection for Low-Cost Vehicle Localization

**DOI:** 10.3390/s18103590

**Published:** 2018-10-22

**Authors:** Kyoungtaek Choi, Jae Kyu Suhr, Ho Gi Jung

**Affiliations:** 1 Department of Electronic Engineering, Korea National University of Transportation, 50 Daehak-ro, Chungju-si 27469, Korea; maninquestion75@gmail.com; 2 School of Intelligent Mechatronics Engineering, Sejong University, 209 Neungdong-ro, Gwangjin-gu, Seoul 05006, Korea; jksuhr@sejong.ac.kr

**Keywords:** low-cost sensor fusion-based precise localization, road sign detection, part-based method, corner detection, real-time processing

## Abstract

In order to overcome the limitations of GNSS/INS and to keep the cost affordable for mass-produced vehicles, a precise localization system fusing the estimated vehicle positions from low-cost GNSS/INS and low-cost perception sensors is being developed. For vehicle position estimation, a perception sensor detects a road facility and uses it as a landmark. For this localization system, this paper proposes a method to detect a road sign as a landmark using a monocular camera whose cost is relatively low compared to other perception sensors. Since the inside pattern and aspect ratio of a road sign are various, the proposed method is based on the part-based approach that detects corners and combines them to detect a road sign. While the recall, precision, and processing time of the state of the art detector based on a convolutional neural network are 99.63%, 98.16%, and 4802 ms respectively, the recall, precision, and processing time of the proposed method are 97.48%, 98.78%, and 66.7 ms, respectively. The detection performance of the proposed method is as good as that of the state of the art detector and its processing time is drastically reduced to be applicable for an embedded system.

## 1. Introduction

Vehicle localization is one of the important components in autonomous driving and advanced driver assistance systems (ADAS) [1]. For vehicle localization, global navigation satellite systems (GNSS) are most widely used [2]. However, the radio signals from satellites are distorted by various causes and these distortions degrade the localization precision. Among the various causes, to overcome atmospheric signal distortion, cooperative positioning (CP) has been developed [3]. CP is an approach that several receivers share the signal distortion information and compensate the distortion cooperatively. Differential GNSS (DGNSS), satellite based augmentation systems (SBAS), ground based augmentation systems (GBAS), and real time kinematic (RTK) are the representative systems in the CP approach. On the other hand, to overcome the diffused reflection of signals on skyscrapers or signal blocking in tunnels, GNSS/INS, which combines GNSS and an inertial navigation system (INS), has been developed [4]. Some GNSS/INS systems can keep their localization error less than the width of a lane even in urban areas but these precise GNSS/INSs are too expensive for mass-produced vehicles. In the case of a low cost INS, since it has a relatively large cumulative position error, the distance maintaining its precision is limited to a short range.

For the above mentioned reasons, map-matching based localization systems have been broadly researched [5]. A map-matching based localization system recognizes landmarks such as buildings or road facilities through perception sensors, estimates the relative pose of a vehicle to the landmark, and estimates the vehicle global position by finding the correspondence of the landmark in a digital map. The map-matching based localization system can be categorized into a 3D feature point-based approach, 2D feature point-based approach, and road facility-based approach according to the perception sensors and types of used landmarks. The 3D feature point based approach collects 3D feature points by scanning the surroundings of an ego-vehicle mainly through light detection and ranging (LIDAR) and estimates the vehicle global position by matching the feature points to the points in a digital map [6,7,8]. Since LIDAR can collect highly precise 3D points, the 3D feature point-based approach can achieve high precise localization. However, in complicated urban areas where there are a lot of temporarily static objects such as parked vehicles or standing pedestrians, the performance of this approach can be degraded. The 2D feature point-based approach is similar to the 3D feature point-based approach except for the fact that it uses an image sensor instead of a depth sensor [9,10,11]. Since the image sensor has enough information to classify objects, the image sensor is advantageous when comparing to LIDAR in excluding points from temporarily static objects. However, this approach is severely affected by seasonal changes and roadside view changes. Both feature point approaches have a shortcoming in that they increase the volume of a digital map to store a lot of feature points. The road facility-based approach recognizes road facilities such as road surface marks or road signs through an image sensor and finds their correspondence in a digital map [12,13,14]. Since most road facilities are strictly maintained by the transport authority and are easily recognized by a driver, they are remarkable and their appearance variation is very small. Furthermore, the information needed to be stored in a digital map is comparatively small.

We have developed a low-cost sensor fusion-based precise localization system that utilizes road surface marks [5]. Our previous work detects lane markings and detects a road surface mark between the lane markings [15]. By utilizing a road surface mark and the relation between a road surface and camera, it estimates the ego-vehicle’s relative position to the road surface mark. However, a road surface mark is often occluded by other vehicles in a congested road. In this case, it cannot achieve the desired precision. This problem can be solved by utilizing additional road facilities not often occluded even in a congested road [16]. As the installation height of a road facility becomes higher, the facility is less occluded by other vehicles. Among the road facilities, a road sign is a good candidate because its installation height is over 5 m and it is well maintained. Therefore, this paper proposes a road sign detection method for vehicle localization.

## 2. Related Works

**Figure 1:**
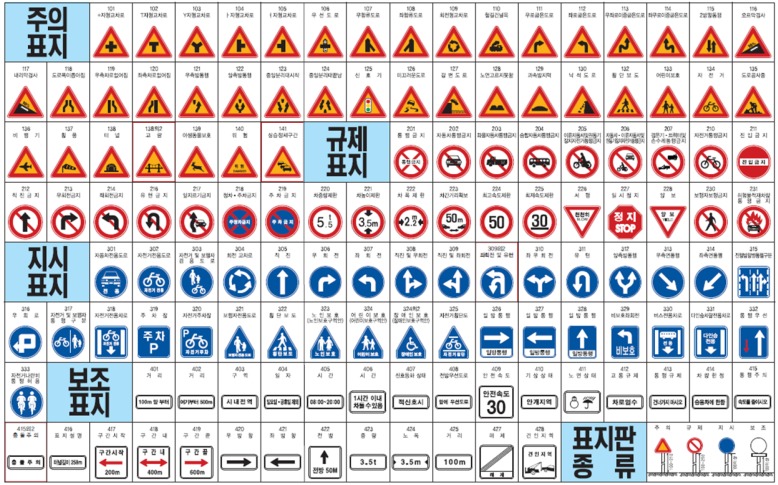
Traffic signs in Korea [31].

Traffic sign detection, similar to the road sign detection handled in this paper, has been researched for several decades [17]. The color of a traffic sign consists of mainly primary colors such as red or yellow, and is limited to either a circular, triangular, or equilateral polygon shape, which is shown in Figure 1. Therefore, the color and shape of a traffic sign are popular features for traffic sign detection. Most color-based methods segment a traffic sign region with color and perform shape detection [18,19]. Color segmentation is done not in the RGB space but in spaces less sensitive to illumination conditions, such as HSV, Otha, or normalized RGB [20]. Since color-based methods are sensitive to illumination conditions and color fading, there are methods depending only on the contour shape [21,22]. Learning-based methods using features extracted not only from its contour but from its wholistic appearance are also popular. Prior to convolutional neural networks (CNN), most learning-based methods have used a cascade classifier inputted with handcrafted features such as Haar, local binary pattern (LBP), integral channel features (ICF), or aggregated channel features (ACF) [23,24,25,26]. Besides these, there are methods using the histogram of gradient (HOG), but these methods are mainly used in the final stage for accurate decisions because of the amount of computation required [27]. Learning-based methods using CNN have been recently studied. Yang et al. propose a two-stage method that segments the region of interest (ROI) with color and detects a traffic sign with a CNN-based classifier [28,29]. Lee et al. propose a detector whose structure is based on a one-stage CNN detector such as a single shot multi-box detector (SSD) [30].

**Figure 2:**
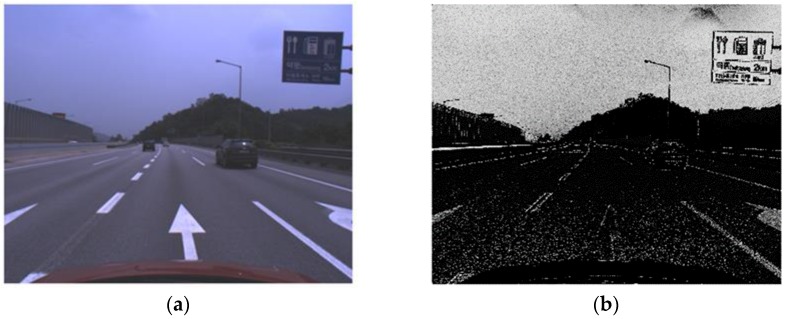
An image of a road sign whose background color is blue (**a**) and the color probability map of the image (**b**) [28].

**Figure 3:**
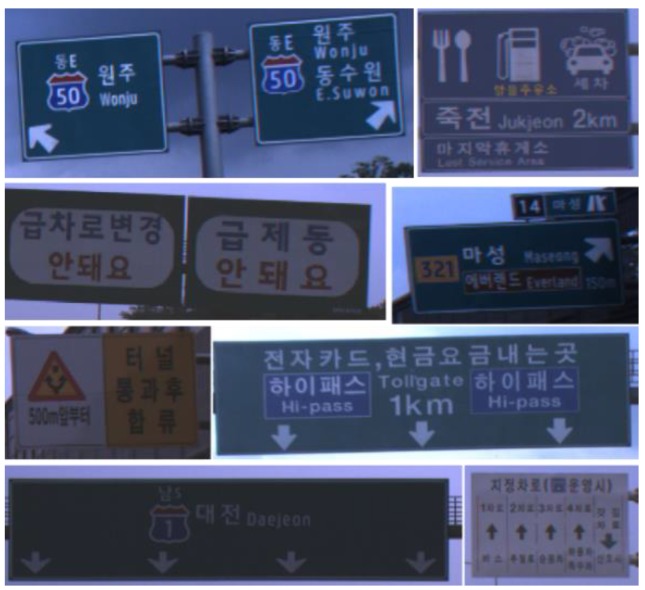
Road signs in Korea.

There are two important differences between road sign detection and traffic sign detection. First, unlike traffic signs, the background colors of a road sign are not composed of primary colors and it is difficult to segment a road sign region with color. For example, the background color of a blue road sign is similar to the color of the sky, which is shown in Figure 2a, and it is difficult to segment a sign with color. Figure 2b is the color probability map for the blue road sign calculated by one of the color-based approaches [28], and the color probability in a road sign is similar to that of the sky. Second, there are a limited number of traffic sign types, which is shown in Figure 1 [31]. Although the size of a traffic sign is changeable according to the speed limit of the road where the sign is installed, its shape and inside pattern are unchangeable. On the other hand, while the shape of the road sign is rectangular, the letters and symbols on it are variable and its aspect ratio is not consistent, which is shown in Figure 3. Due to this difference, the traffic sign detection methods that detect specific shapes such as equilateral polygons or extract features from the wholistic appearance, are not effective for road sign detection.

This paper proposes a part-based road sign detection method that detects the four corners of a road sign and detects the sign by combining these corners. For vehicle localization, the recognition of a road sign is not necessary since the installation interval of a road sign is much longer than the GNSS/INS error range, and therefore it is not possible that a detected road sign will falsely correspond with the sign in a map. The proposed method is less sensitive to illumination conditions since it does not execute color segmentation. In addition, it is irrelevant to the pattern variation inside a road sign and is less affected by the aspect ratio variation. The proposed method directly detects the four corners through the learning-based method instead of extracting the four boundary lines of a road sign and finding intersections of the lines. The reasons are that the boundary lines are often partially occluded and the intersection is very sensitive to the directional error of the lines. In the part-based approach, there is a method using an aggregated channel feature (ACF) detector [32,33] and our previous method using a Viola-Jones (VJ) detector [34]. Both of these previous methods have shown excellent performance, but the amount of computation is too large to operate in real-time on a vehicle. In this paper, we upgrade our previous method to operate in real time by setting up the region of interest (ROI) for the corner detection through features from the accelerated segment test (FAST) [35]. Furthermore, the comparative experiment in this paper proves that the detection performance of the proposed method is as good as that of YOLOv3. YOLOv3 is one of the state of the art CNN-based detectors and is famous thanks to its high detection performance and high throughput rate [36]. The processing time of the proposed method is remarkably reduced when compared to previous works.

## 3. System Overview

**Figure 4:**
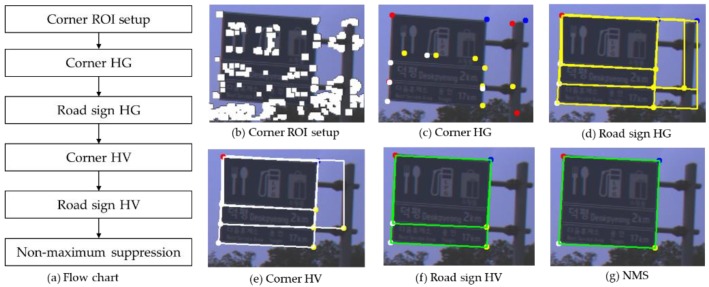
System overview.

The proposed method consists of six steps, which is shown in Figure 4a. The corner ROI setup extracts FAST corners and makes a corner ROI map by applying a dilation filter around the FAST corners, which is shown in Figure 4b. The corner ROI setup is described in detail in Section 4.

In the proposed method, a road sign and its corners are detected through a two stage method consisting of hypothesis generation (HG) and hypothesis verification (HV) [37]. The corner HG generates the hypotheses of four type corners within the corner ROI by using VJ detectors whose feature is a local binary pattern (LBP) [38,39]. The corner HG uses four VJ detectors, which are trained individually for four corner types which are left-top, right-top, left-bottom, and right-bottom corners depicted as red, blue, white, and yellow dots in Figure 4g. How to train four VJ detectors and their parameters, is described in detail in the Appendix A.

After the corner HG, road sign hypotheses are generated from the combination of four type corner hypotheses, which satisfy the geometric constraints of road signs. In this step, many false corner hypotheses may be rejected and the remaining false corner hypotheses are filtered again in the corner hypothesis verification (HV). The road sign HG is described in detail in Section 5.

The corner HV utilizes a support vector machine (SVM) whose feature is a histogram of gradient (HOG) [40,41]. The road sign HG is very simple and it can reject efficiently the false corner hypotheses comparing to the corner HV. Therefore, the corner HV after the sign HG is more efficient than the reverse order. The road sign HV resizes image patches extracted from the sign hypotheses and verifies the patches by using HOG-SVM. How to train the corner HV and the road sign HV, and their parameters, is described in detail in the Appendix A. 

Lastly, since the verified road sign hypotheses might overlap, they are integrated as one in the non-maximum suppression (NMS) step shown in Figure 4g. The NMS is described in detail in Section 6.

## 4. Corner ROI Setup

**Figure 5:**
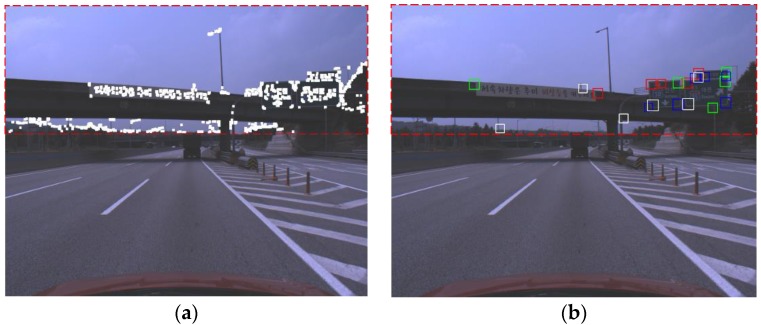
Example of an ROI for corner HG and corner hypotheses. (**a**) ROI for corner HG, (**b**) corner hypotheses generated by a VJ detector.

In this paper, in order to reduce the processing time, we use a general corner detector to reduce the region of interest (ROI) of the VJ detector rather than directly use it for the corner detection. In order to detect corners on a road sign, general corner detectors may be applied [35,42,43,44]. However, general corner detectors have a very low recall and precision compared to the VJ detector trained only for road sign corners. The 2nd and 3rd columns in Table 1 are the results of adjusting the threshold of general corner detectors to detect 1500 and 150 corners, respectively. Adjusting the threshold lower causes many falsely detected corners, as shown in the 2nd column of Table 1 and the high threshold causes many missing corners on a road sign, which is shown in the 3rd column of Table 1. Moreover, adjusting the threshold even lower until it misses some sign corners will result in many more false corners when compared to the VJ detector results in Figure 5b. 

The VJ detector has a higher recall and precision than general corner detectors, but it is difficult to operate in real time on a vehicle because of the large amount of computation required. Since the VJ detector is a cascade type detector, positions other than the corners are mostly filtered at the early stages. However, as the VJ detector applies multiple weak classifiers in each stage and checks multiple patches to consider the object scale variation, it does take a certain amount of time. In addition, since the proposed method applies an individual VJ detector for each corner type, the processing time is four times longer than when a single VJ detector is applied. We tried to detect four types of corners with a single VJ detector. However, when compared to applying an individual detector for each corner type, the recall was similar but the precision decreased by about 5%. In addition, in this case, the type of corner cannot be known and the amount of computation in the following steps (the road sign HG and corner HV) will be increased.

In this paper, in order to reduce the region of interest (ROI) of the VJ detector, we use FAST that is known to have the smallest amount of computation and to have high repeatability among general corner detectors [35]. We adjust the threshold of FAST as low as possible to make the recall almost 100% and to maintain high precision at the same time. In Figure 5a, the white blobs are the results of applying the dilation filter around the FAST corners and the four VJ detectors generate corner hypotheses only on these blobs. Since there is a difference between the corner positions detected by FAST and the VJ detector, this paper applies the dilation filter around the FAST corners. According to the installation guide for a road sign on a Korean highway, the sign should be installed at a height of 5 m above a road surface [45]. Therefore, road signs are detected not in the entire region but only in the upper part (depicted as a red dotted box) of an image, which is shown in Figure 5a. Part-based road sign detection generates corner hypotheses for four corners, as shown in Figure 5b. In Figure 5b, the left-top, right-top, right-bottom, and left-bottom corner hypotheses generated by the VJ detector are depicted as red, green, blue, and white boxes, respectively. 

## 5. Road Sign Hypothesis Generation and Verification

**Figure 6:**
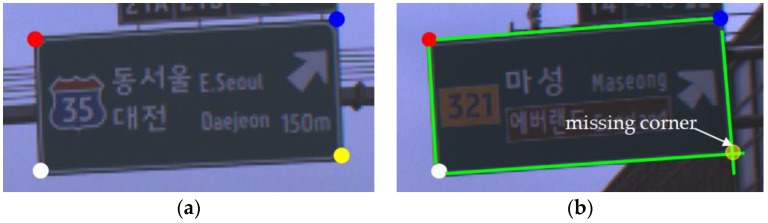
Examples of road sign hypotheses. (**a**) four corner combination, (**b**) three corner combination.

**Figure 7:**
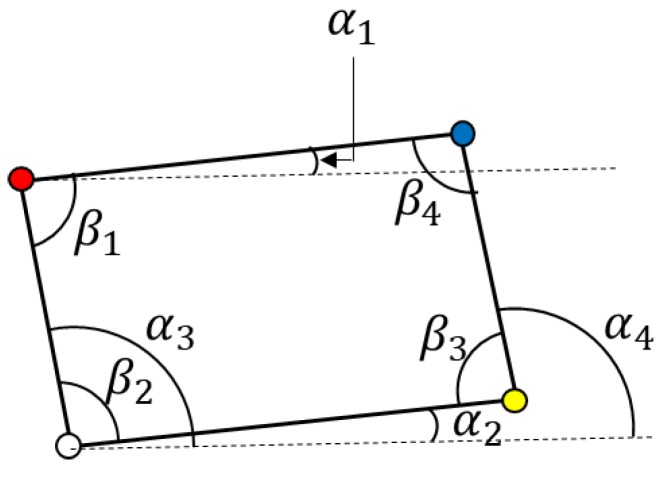
Geometric constraints for road sign HG.

After corner hypotheses are generated by four VJ detectors, three or four corner hypotheses are combined to generate a road sign hypothesis. In the case of combining four corners, a road sign hypothesis is generated as a quadrangle consisting of four different corner types, which is shown in Figure 6a. When missing one corner on a road sign, a road sign hypothesis is generated as a parallelogram made with three different corner types and the missing corner is replaced with a vertex of a parallelogram, as shown in Figure 6b. Among quadrangles or parallelograms, the combinations are satisfied with the geometric constraints and will exist as road sign hypotheses. The geometric constraints are the ranges of eight angles shown in Figure 7 and the aspect ratio. The ranges estimated from the statistics of the training data set are described in the Appendix A. In addition, if the installation height of a road sign is known, the physical size of a road sign can be estimated and it can be used to filter out false road sign hypotheses. According to the installation guide [45], the lowest side of a road sign should be at a height of 5 m above a road surface. However, it is impossible to follow the guide exactly and the real installation height of a road sign in the training data set is from 4.5 m to 6 m. Therefore, instead of applying the estimated size strictly, we filter out only the road sign hypotheses whose estimated height and width are smaller than 1 m.

**Figure 8:**
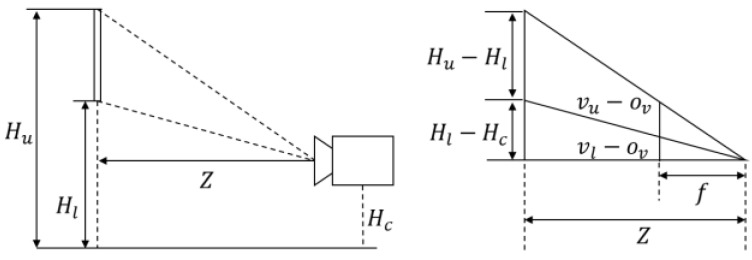
Side view of road sign, virtual camera, and road surface.

**Figure 9:**
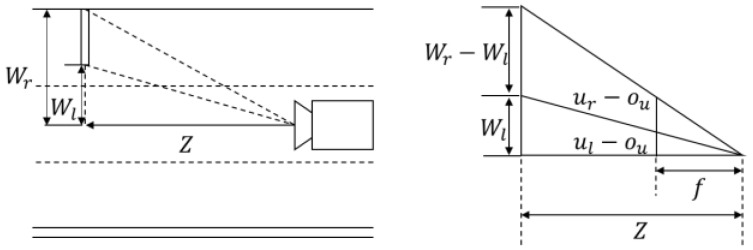
Top-view of road sign, virtual camera and road surface.

The physical size of a road sign is estimated as follows. The camera extrinsic parameters with respect to the vehicle coordinate system and the camera intrinsic parameters are estimated by the offline calibration in advance. After the calibration, the virtual camera coordinate system whose optical axis (*z*-axis) is parallel to the vehicle’s traveling direction and whose image plane is perpendicular to the ground can be set up. Figure 8 shows the side view of a road sign and the virtual camera. In Figure 8, the distance Z from the camera to the road sign is calculated by using the equation below.

(1) $$Z=\frac{f\left(H_{l}-H_{c}\right)}{\left(v_{l}-o_{v}\right)}$$

where, $H_{l}$ and $H_{c}$ are the installation height of a road sign and the camera height, f and $o_{v}$ are the camera focal length and the vertical coordinates of the camera principle point, and $v_{l}$ is the vertical image coordinates of a mid-point of two lower corners on a road sign. $H_{l}$ is assumed to be 5 m by referencing the guide, and the distance Z is estimated. From the estimated Z, the physical height H and width W of a road sign are calculated with the equations below.

(2) $$H=\left(H_{u}-H_{l}\right)=\frac{Z\left(v_{u}-v_{l}\right)}{f}$$

(3) $$W=\left(w_{r}-w_{l}\right)=\frac{Z\left(u_{r}-u_{l}\right)}{f}$$

where $u_{r}$ and $u_{l}$ are the horizontal image coordinates of mid-points of two left corners and two right corners on a road sign. Figure 9 explains the physical meaning of Equation (3).

After the road sign hypotheses are generated by considering the geometric constraints, each corner on the hypotheses are verified by a corner type-specific SVM classifier whose feature vector is HOG. The road sign where all of corners are verified as positives, is corrobarated by an SVM classifier in which the HOG feature is extracted from the whole of the road sign.

## 6. Non-Maximum Suppression

**Figure 10:**
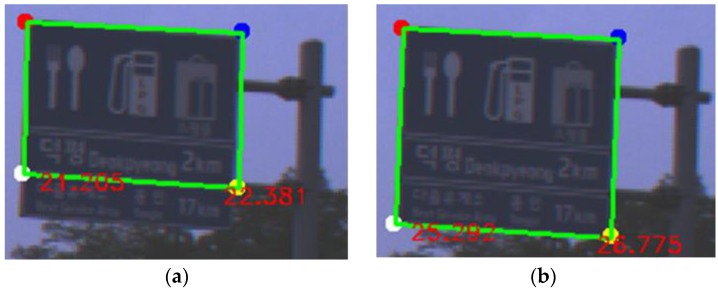
The effect of NMS considering the height of the road sign ((**a**): considering only the road sign HV score, (**b**): considering the height as well).

The NMS selects the optimal one among the road sign hypotheses that overlap. The degree of overlap is calculated as the intersection over the union (IOU). If the road sign HV score difference or width difference between the overlapped road signs is over a threshold, the NMS selects the road sign where the HV score is higher than the other’s. Otherwise, a small additional road sign is assumed to be attached to the main road sign, which is shown in Figure 10, and the NMS selects the road sign whose height is larger than the other sign’s height. As the size of a road sign becomes bigger, the localization error by using this sign becomes smaller. Therefore, the NMS selects the bigger one among the overlapped signs.

## 7. Experiments

### 7.1. Experimental Database

**Figure 11:**
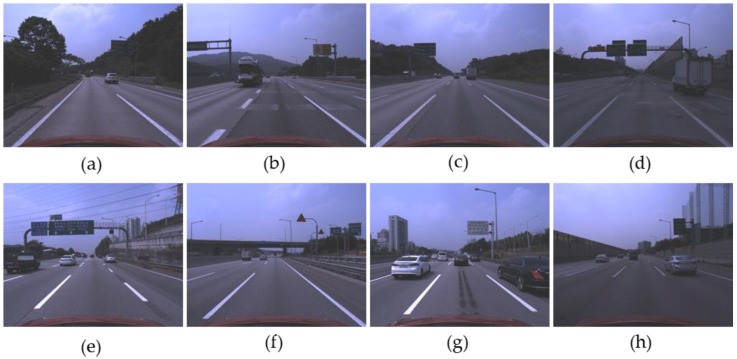
Examples of the experimental database. (**a**) low contrast between a road sign and background, (**b**) a road sign consisting of two colors, (**c**) low contrast between a road sign and background, (**d**) general road signs, (**e**) a road sign having large aspect ratio, (**f**) a road sign which is far from a camera, (**g**) a white road sign, (**h**) a road sign having small aspect ratio.

The database used in this paper is explained in Table 2. The database was collected within about a 42 km range from the Seoul toll gate to the Hobeop junction of Yeongdong highway in South Korea. In Table 2, DB 1 is used only to train the VJ corner detector, and DB 2 is used for all training phases including the VJ corner detector, corner HV, road sign HV, and the statistics of road sign HG. DB 3 is used to evaluate each algorithm step. All three DBs were collected by the same camera but the type of vehicle used for collection in DB 1 is different to that in collecting DB 2 and DB 3. DB 2 and DB 3 were collected from different direction lanes of the expressway. The different direction lanes in the expressway are divided by a central reservation and they may be considered as different roads. The image resolution is 1280 × 1024. Figure 11 shows sample images in the experimental database. 

The experimental database includes the various kinds of road sign and various backgrounds such as forests, bridges, and soundproofing walls. Figure 11a,c show the case that the contrast between a road sign and background is very low. Figure 11b shows the road sign whose background is painted by two colors. Figure 11f shows the road sign far from an ego-vehicle. Figure 11e,h show the road signs with various aspect ratios. We define the maximum detection distance as 30 m. For evaluation, we assume the installation height of a road sign as 5 m and select the images including the road signs whose estimated distances from an ego-vehicle are within 30 m.

### 7.2. Experimental Results

As shown in Table 3, we compare three methods: the proposed method without the FAST corner ROI, the proposed method with the FAST corner ROI, and YOLOv3 using the convolutional neural network (CNN) [36]. The proposed method is one of the part-based approaches but YOLOv3 is trained to detect a whole body of a road sign at once and is one of the appearance-based approaches. Therefore, YOLOv3 does not have several intermediate steps unlike the proposed method, and only its final performance is described in Table 3. The H/W spec for the experiment follows as: CPU (i7-7700@3.6GHz), OS (windows 10), and RAM (16 GB). The method without the FAST corner ROI sets the upper part of an image whose vertical coordinate is under 450 as the ROI for the corner HG. In Table 3, the corner HG processing time of the proposed method is the summation time of the corner ROI setup and the corner HG. In the corner HG, the VJ detector is our modified version of the OpenCV library to operate in a single thread [46]. In order to evaluate the corner detection performance, if the distance between a corner ground truth (GT) and a detected corner is less than 10 pixels, the detected corner is considered as a true positive (TP) and otherwise as a false positive (FP). In the case of a road sign, if the IOU between a detected road sign and a road sign GT is over 0.5, the detected road sign is considered as a TP and otherwise as a FP. The recall and the precision for the corner level are evaluated only in the corner HG step and the performances in the other steps are evaluated for the road sign level. 

In the corner HG step, it is important to keep the recall of road sign corners high. In the case of using the FAST corner ROI, while the recall is rarely degraded, the processing time is reduced drastically. The experimental results show that the proposed method can reduce the processing time of the corner HG up to four times by using the FAST corner ROI. The recall in the sign HG step is slightly improved because the road sign hypothesis with one missing corner can be also generated through the parallelogram relation. Furthermore, the low precision in the corner HG indicates that there are a lot of falsely detected corners. Nevertheless, the sign HG which takes just 2.5 ms significantly improves the precision and this shows that a lot of falsely detected corners can be effectively removed through geometric constraints. The precision improvement by the corner HV is small but the improvement by the sign HV is large. This indicates that even if there are few false corners, there may be a lot of false corner combinations that satisfy the geometric constraints. The final performance after passing the sign NMS step shows greater improved precision and this indicates that a lot of road sign hypotheses overlap.

**Figure 12:**
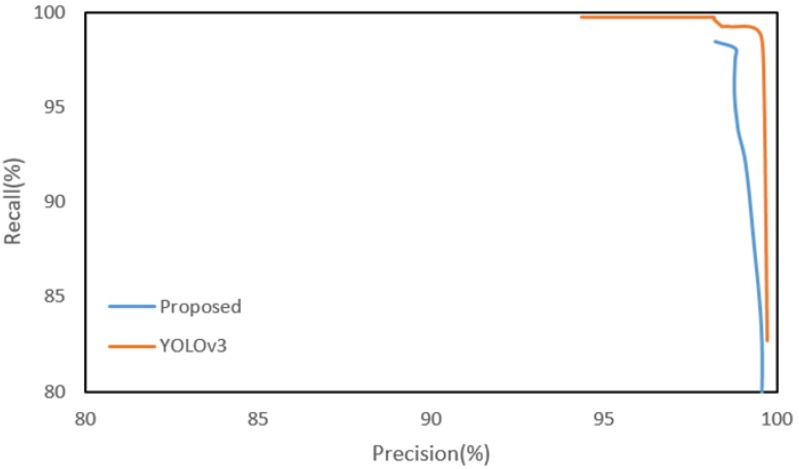
Recall-precision curves of the proposed method (with FAST corner ROI) and YOLOv3.

The final road sign recall of the proposed method is 97.48% or about 2% lower than YOLOv3, but the precision is 98.78%, which is better than YOLOv3. Figure 12 shows the recall-precision curves of the proposed method and YOLOv3. The curve of the proposed method is generated by adjusting only the SVM threshold of the road sign HV. Since some of the true corners may be missed in the steps prior to the road sign HV, the recall of the proposed method cannot reach 100% by adjusting only the SVM threshold. Although the detection performance of the proposed method is slightly lower than YOLOv3, the proposed method may be more effective than YOLOv3 in a vehicle localization system when considering real time processing. The proposed method takes a total of about 66.7 ms and can process 15 frames per second. However, YOLOv3 takes a total of about 4802 ms on the CPU even if the input image resolution is reduced to a quarter (640 × 512). Yet, the processing time for all three methods can be significantly reduced through a high-performance parallel processing H/W like the GPU, but the price and operating conditions of the GPU have not yet met the requirements of a vehicle. 

**Figure 13:**
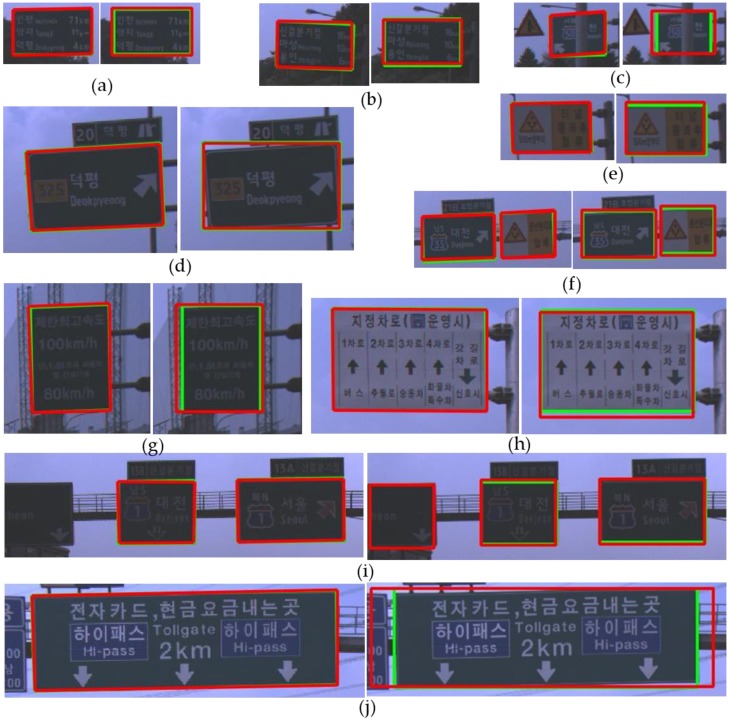
Comparison of the true positive detections between the proposed method and YOLOv3 (left: the proposed method, right: YOLOv3). (**a**) low contrast between a road sign and background, (**b**) low contrast between a road sign and background, (**c**) a partially occluded road sign, (**d**) a rotated road sign, (**e**) a road sign consisting of two colors, (**f**) two road signs whose kinds are different, (**g**) a road sign whose aspect ratio is small, (**h**) a white road sign, (**i**) a road sign partially observed, (**j**) a road sign whose aspect ratio is large.

**Figure 14:**
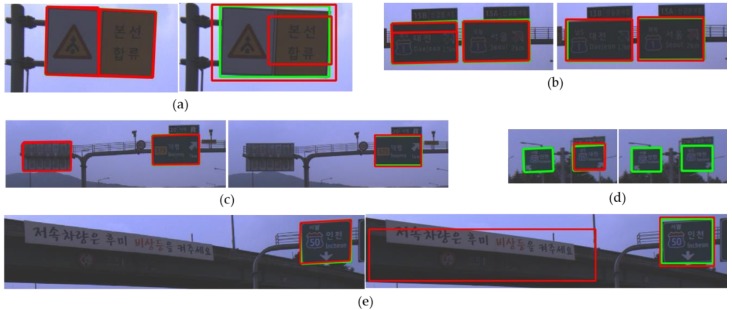
Comparison of the detection errors between the proposed method and YOLOv3 (left: the proposed method, right: YOLOv3). (**a**) a road sign consisting of two colors, (**b**) a road sign not precisely detected by the proposed method, (**c**) a road sign falsely detected by the proposed method, (**d**) road signs not detected, (**e**) a road sign falsely detected by YOLOv3.

Figure 13 shows good detection results of both the proposed method and YOLOv3. In Figure 13, a red box depicts a detection result and a green box depicts a ground truth. In the sub-figures of Figure 13, the left side is the result of the proposed method and the right side is the result of YOLOv3. Even in cases that the contrast between a road sign and background is too low to find the boundary clearly between them as shown in Figure 13a,b, both the proposed method and YOLO v3 detect a road sign successfully. Figure 13c shows that both methods can detect a partially occluded road sign. Although YOLOv3 adopts an appearance-based approach, this extracts many complicated features through deep CNN and can detect a partially occluded road sign. On the other hand, the proposed method uses relatively simple hand-craft features. However, due to the adoption of the part-based approach, the proposed method can detect also a partially occluded road sign if more than three corners of the road sign are detected. As shown in Figure 13d, the proposed method can find the boundary of the rotated road sign more accurately than YOLOv3 because the proposed method detects the corners of the sign. In the localization system, it is important to accurately detect the image points corresponding to the reference points of a road sign stored in the map. In this view point, the part-based approach such as the proposed method is more advantageous than the appearance-based approach. Figure 13e,f show that both methods detect a road sign whose background is divided into two regions. However, Figure 14a shows that this kind of a road sign may be sometimes detected as two separate ones by both methods. Figure 13g–j show that road signs whose aspect ratios and kinds are different can be detected well by both methods.

Figure 14 shows examples where there is an error in at least one of the detection results of both methods. The proposed method detects incorrectly one road sign as two separate ones which is shown in Figure 14a. However, this error can be solved by applying the NMS between different corner types. Figure 14b shows an example that the proposed method fails to integrate the overlapped road sign hypotheses correctly because of the tight conditions of the road sign NMS. The proposed method is based on the part-based approach and the method does not utilize the inside patterns on a road sign as detection features. As a result, the proposed method can unintentionally detect a road sign not facing an ego-vehicle, which is shown in Figure 14c. However, YOLOv3 can filter out these unintended detections by utilizing the inside patterns of the sign. On the other hand, this utilization of the inside patterns causes the side effect that the letter patterns on the background can be falsely detected as a road sign, which is shown in Figure 14e. Figure 14d shows the detection failures of road signs far from an ego-vehicle. Only the proposed method detected one of two road signs in the far distance. Yet, if the processing time is no matter, YOLOv3 may detect the road signs in the far distance by increasing the input image resolution.

## 8. Conclusions

This paper proposes a road sign detection method for a low-cost sensor fusion-based precise localization system. The proposed method focuses on the real-time operation in embedded systems such as current mass produced vehicles not equipping any expensive parallel processing hardware. The proposed method reduces the processing time significantly to 66.7 ms by using the FAST corner ROI. Furthermore, the detection performances of the proposed method are a recall of 97.48% and a precision 98.78%. These performances are comparable to those of a CNN-based representative object detector. As the distance from the detected road sign to the camera becomes shorter, the estimated vehicle position by using the sign becomes more precise. Therefore, by referring the frame work of the reference [26], we will add the corner tracking module in order to detect the road sign where the partial area is out of a camera FOV. In addition, we plan to use the information from a digital map to reduce the ROI and increase the precision like our traffic light detection [47]. We plan to upgrade our vehicle localization system [5] by using a road sign and other road facilities together such as a lane mark, a road mark, and a traffic sign. 

## Figures and Tables

**Table 1:** Comparison of general corner detectors.

Method	Number of Corners (1500)	Number of Corners (150)
FAST [35]	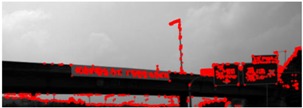	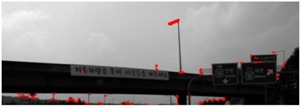
Harris [42]	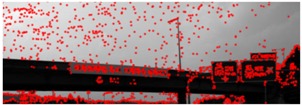	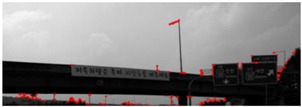
Shi [43]	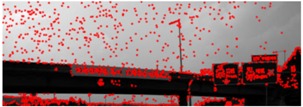	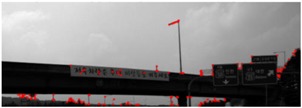
BRISK [44]	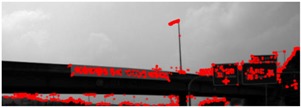	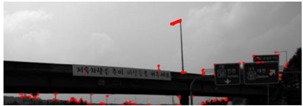

**Table 2:** Experimental database.

DB No.	No of Images	Frame Rate	No of Road Signs	Usage
1	22,121	15	1371	Training VJ corner detector
2	40,076	20	1093	All training phases
3	32,345	20	832	All test phases

**Table 3:** Experimental results.

Steps	Without FAST Corner ROI			With FAST Corner ROI			YOLOv3 [36]		
	Recall (%)	Precision (%)	Time (ms)	Recall (%)	Precision (%)	Time (ms)	Recall (%)	Precision (%)	Time (ms)
Corner HG	97.27	22.48	183.7	97.20	19.56	46.6			
Sign HG	98.80	51.89	185.1	99.04	53.13	48.2			
Corner HV	98.80	55.76	186.3	98.92	55.68	49.5			
Sign HV	97.83	93.03	202.3	97.72	92.28	66.5			
Final	97.59	99.39	202.8	97.48	98.78	66.7	99.63	98.16	4802

**Figure A1:**
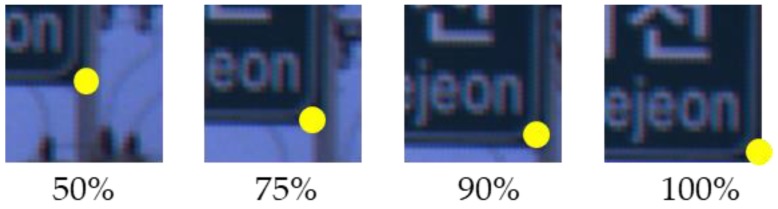
Side length ratio in the training corner patch.

## Appendix A

The parameters of each algorithm step set by the experiment with the training database are summarized in Table A2. Depending on the FAST threshold and the dilation filter size, there is a trade-off between the recall and the processing time. In order to keep the recall of the corner HG as high as possible, we adjust the FAST threshold to a very low level. When the FAST threshold is 10, 98.53% of road sign corners are within 10 pixels from FAST corners. Therefore, we set the FAST threshold as 10. Afterward the corner HG performance was analyzed while changing the dilation filter size with the training DB, which is shown in Table A1. The dilation filter size is set as 9 by considering the processing time and detection performance.

**Table A1:** Corner HG performance according to the dilation filter size with the training DB.

Corner HG Performance	Filter Size						
	3	5	7	9	11	13	15
Time (ms)	35.48	39.66	42.06	46.65	49.09	53.00	54.60
Recall	89.50%	94.40%	96.50%	97.60%	98.00%	98.30%	98.40%
Precision	44.10%	33.20%	27.60%	23.40%	21.70%	20.40%	20.00%

The side length ratio in the corner HG of Table A2 indicates the ratio between the side lengths of the foreground and the training patch which is shown in Figure A1. The ratio is set to 75% having the best result among 50%, 75%, 90%, and 100% through the experiment. There is an individual VJ corner detector for each corner type. To generate the training patches of the VJ detector, the square image patch whose height is 12% of a road sign height is extracted around a corner and augmented patches are generated by scaling the patch size 0.9 and 1.1 times. Then, all of the training patches are resized to 24 × 24. About 4600 positive and 8000 negative samples are used to train each type of corner detector.

The geometric constraints in the road sign HG are from the statistics of DB 2 and they are summarized in Table A3. The training samples for the corner HV are the rest after removing the training samples for the corner HG that are hard to be classified manually. The corner HV are also individually trained for each corner type, and the numbers of positive and negative training samples are from 1500 to 2400 and from 500 to 2000 respectively. To train the road sign HV, 1136 positive and 1710 negative samples are used. The HOG and SVM parameters of the road sign HV, except the patch size, are the same as those of the corner HV.

**Table A2:** Parameters for each algorithm step.

Algorithm Step	Parameters	Value
Corner ROI setup	FAST threshold	10
	Dilation filter size	9
Corner HG	Feature	Local Binary Pattern
	Training patch size	24 × 24
	Side length ratio in training patch	75%
	Detecting patch size range	24 × 24~56 × 56
	Scale factor	1.1
Corner HV	HOG Patch size	24 × 24
	Block size	16
	Stride	8
	Cell size	8
	Number of bins	9
	SVM threshold	−0.3
Road sign HV	HOG Patch size	120 × 72
	Other parameters	Same as the corner HV
Road sign NMS	IOU threshold	0.3

**Table A3:** Valid value range of the geometric constraints.

	$\alpha_{1}$	$\alpha_{2}$	$\alpha_{3}$	$\alpha_{4}$	$\beta_{1}$	$\beta_{2}$	$\beta_{3}$	$\beta_{4}$	Aspect Ratio
Min	−6.1°	−4.7°	86.8°	85.0°	−94.2°	88.0°	−93.7°	83.9°	0.18
Max	5.7°	4.5°	94.0°	94.4°	−85.5°	93.3°	−87.2°	93.5°	1.4
